# Dataset on preparation of the phosphorylated counterparts of *a Momordica charantia* protein for studying antifungal activities against susceptible dose-dependent *C. albicans* to antimycotics

**DOI:** 10.1016/j.dib.2017.09.041

**Published:** 2017-09-23

**Authors:** Yuanbiao Qiao, Li Song, Chenchen Zhu, Qian Wang, Tianyan Guo, Yanhua Yan, Qingshan Li

**Affiliations:** Graduate Institute of Pharmaceutical Chemistry, Luliang University, Shanxi, PR China

**Keywords:** *in vitro* phosphorylation, *Momordica charantia* protein, Protein kinase catalysis, Antifungal susceptibility testing, *C. albicans*

## Abstract

The data presented here are related to a research article entitled “Development of a phosphorylated *Momordica charantia* protein system for inhibiting susceptible dose-dependent *C. albicans* to available antimycotics: An allosteric regulation of protein” (Qiao et al., 2017) [1]. The data set includes three portions: (1) a relationship between reaction velocities of protein phosphorylation as a function of the substrate concentrations, determined in enzymatic reactions in aid of protein kinases; (2) a result of antifungal susceptibility testing of *C. albicans* after it is selected in antimycotics; and (3) a comparison of protein expression in the susceptible dose-dependent fungus relative to the wild *C. albicans*. In the first portion, the relationship of reaction velocities and substrate concentrations is expressed as an output from the inverse variation model. All data and analyses are made publicly available and citied in the research article using a style for *the Data in Brief.*

**Specifications Table**

**Table:** 

Subject area	*Biochemistry and Microbiology*
More specific subject area	*Enzyme kinetics and antifungal susceptibility*
Type of data	*Tables*
How data was acquired	*For determinations of the Momordica charantia protein phosphorylated counterparts, we follow experimental steps for alternatively sampling along reaction kinetic process, treating samples via diafiltration and heat-digestion, and then testing their phosphate amounts on a microscope spectrophotometer via a phosphomolybdenum blue spectrophotometric method; antifungal susceptibility testing of C. albicans is followed by a McFarland equivalence standard method according to the guidelines of Antifungal Susceptibility Testing Subcommittee of the Clinical and Laboratory Standards Institute in the USA; and for protein expression assays, the dose-dependent susceptible C. albicans is cultured in the liquid media with and without a load of antimycotics. After collection of fungal isolates, they are ultrasonically disintegrated to release the proteases expressed. The protein expression is determined by spectroscopy using a coomassie brilliant blue R-250 staining method.*
Data format	*Analyzed, Raw*
Experimental factors	*Heat-digestion of the Momordica charantia protein phosphorylated counterparts at 120* *°C for 0.5* *h.*
	*Antifungal susceptibility testing of C. albicans lineages after selecting on the Sabouraud's dextrose agar loaded with antimycotics.*
Experimental features	*The protein phosphorylated counterparts were siphoned off at a reaction interval of 0.5* *h, diluted by 1:75 and treated by diafiltration (Cut-off: 3.5 kD).*
	*Dose-dependent of susceptibilities in C. albicans were acquired through gradual selection to ketoconazole, econazole, miconazole, 5-flurocytosine, nystatin and amphotericin B respectively at an increasing dosage set of individuals.*
Data source location	*Graduate Institute of Pharmaceutical Chemistry, Luliang University, Luliang City, 033001, Shanxi, PR China.*
Data accessibility	*The data are available with this article.*

**Value of the data**

• A mathematical statistics of reaction velocities and substrate concentrations yields the apparent Michaelis-Menten constants and maximal velocity values of the *Momordica charantia* protein phosphorylation with different substrates, while catalyzed by cyclin-dependent kinase 1, protein kinase A and protein kinase C respectively. Also, the result allows quantitative construction of a substrate-free molecular biopharmaceutics system of this protein.
• Antifungal susceptibility testing of *C. albicans* expands on the laboratory fungal strain lineages to be studied by researchers. Fungus, in the dose-dependent susceptibility profiles against the antimycotics of azole, fluorinated pyrimidine analogue, lipopeptide and generic families, is a hazardous material towards candidemia infectious. This kind of problems is however unresolved.
• A study on protein expression of *C. albicans* in the dose-dependent susceptibility profiles gives evidence for a strong selection potential of phenotypes to antimycotics. The result will help to elucidate an antifungal susceptibility mechanism in a proteomic level.

## 1 Data

The dataset of this article provides information on enzymatic kinetics for *in vitro* phosphorylation reactions of a *Momordica charantia* protein with the 5′-adenylic, guanidylic, cytidylic and uridylic acids and cyclic adenosine triphosphate substrates, in aid of catalysis of cyclin-dependent kinase 1, protein kinase A and protein kinase C respectively (Table 1). Also, information on antifungal susceptibility testing of the laboratory *C. albicans* lineages (Table 2) and intracellular protease expression of fungal cells (Table 3) is provided. Detailed interpretation is referred to a Ref. [1].

**Table 1:** Enzymatic parameters of *Momordica charantia* protein phosphorylation by different substrates.

Protein kinase sort	Substrate type	Substrate concentration, ×10^4^ μM^−1^	Reaction velocity^a^, (μM/min)^−1^
Cyclin-dependent kinase 1	5′-adenylic acid	1.02	0.03
		1.51	0.05
		2.23	0.06
		3.01	0.08
		3.80	0.10
		1.16	0.04
	5′-guanidylic acid	1.36	0.05
		2.02	0.06
		2.71	0.08
		3.3	0.10
	5′-cytidylic acid	0.30	0.02
		0.80	0.05
		1.65	0.06
		2.81	0.09
		4.30	0.14
	5′-uridylic acid	1.05	0.05
		1.23	0.05
		1.84	0.07
		2.45	0.10
		3.50	0.120
	Cyclic adenosine triphosphate	1.00	0.04
		1.70	0.08
		2.45	0.12
		3.00	0.14
		3.50	0.15
Protein kinase A	Cyclic adenosine triphosphate	0.04	0.06
		0.08	0.07
		0.10	0.13
		0.14	0.15
		0.15	0.16
Protein kinase C	Cyclic adenosine triphosphate	0.83	0.04
		1.36	0.08
		2.32	0.10
		2.88	0.14
		3.03	0.15

*Notes:* An apparent concentration of the *Momordica charantia* protein is 0.165 μM, and the substrate concentrations are 0.165, 0.169, 0.173, 0.177 and 0.181 mM. Mixture is diluted by 1:75 and determined at a 0.5-h interval of reactions;

a Significance codes (*p*)≤0.05.

**Table 2:** Identification and antifungal susceptibility testing of the *C. albicans* strain lineages to antimycotics.

Reagent	SDD profiles				E-testing		
	Microdilution, µg ml^−^^1^	Plate count^a^, No.·cm^−^^2^	Colony color	Isolate No.	SDD isolates percentage, %	MIC^b^, µg ml^−1^	*p*
None	–	6.3	White	33	3.3	–	–
5-FC	21.33	5.2	White	18	62.5	2.33–3.20	0.05
	42.67	2.8		12	87.5		
	64.00	1.2		8	100.0		
NY	1.33	3.3	Yellow	20	55.56	0.26–0.33	<0.01
	2.66	1.6		14	88.9		
	4.00	0.5		9	100.0		
EZ	4.00	4.6	Yellow	22	57.1	0.88–1.03	0.03
	8.00	2.8		10	85.7		
	16.00	0.3		7	100.0		
AMB	2.67	2.6	White	13	60.0	0.54–0.62	0.02
	5.33	0.8		8	80.0		
	8.00	0.3		5	100.0		
MC	3.77	5.0	White	17	57.1	0.66–0.82	0.03
	7.53	2.1		10	71.4		
	11.30	0.6		7	85.7		
KC	21.33	4.8	Yellow	18	62.5	3.04–3.88	0.05
	42.67	1.6		9	87.5		
	64.00	0.4		5	100.0		

*Note:* 5-FC = 5-flucytosine, NY = nystatin, EZ = econazole, AMB = amphotericin B, MC = miconazole, and KC = ketoconazole; SDD = susceptible dose-dependent;

a The 48-h incubation of aerobic growth, a 0.5 McFarland standard fungal suspension is diluted by 100 folds, and 0.1 ml inoculum is plated.

b Average of triplicate assays after the 24-h culture in liquid media.

**Table 3:** Protein expression of the susceptible dose-dependent *C. albicans* strain lineages.

Strain	Medium	Fungal growth, g l^−1^	Protein expression^a^, mg g^−^^1^
Wild *C. albicans*	SDA^b^	1.63	0.65
5-FC SDD *C. albicans*	SDA^b^	1.70	0.68
	SDA^c^	1.26	0.81
NY SDD *C. albicans*	SDA^b^	1.70	0.73
	SDA^c^	1.38	0.83
EZ SDD *C. albicans*	SDA^b^	1.65	0.69
	SDA^c^	1.18	0.84
AMB SDD *C. albicans*	SDA^b^	1.42	0.77
	SDA^c^	0.88	0.90
MC SDD *C. albicans*	SDA^b^	1.45	0.82
	SDA^c^	1.04	1.03
KC SDD *C. albicans*	SDA^b^	1.74	0.88
	SDA^c^	1.32	1.16

*Note:* F-FC = 5-flucytosine, NY = nystatin, EZ = econazole, AMB = amphotericin B, MC = miconazole, and KC = ketoconazole; SDD = susceptible dose-dependent; SDA = Sabouraud's dextrose agar;

a Results after the 24-h culture, for an average in triplicates.

b No antimycotics.

c A load of 42.66, 2.66, 8.00, 5.34, 7.54 and 42.66 μg ml^−1^ 5-FC, NY, EZ, AMB, MC and KC respectively.

## 2 Material and methods

### 2.1 Reaction kinetics of protein phosphorylation

A previous *Momordica charantia* protein [2] is dissolved in phosphate buffer solution (PBS, pH 6.86). An aliquot of 100-µl protein solution (0.33 mM) is mixed separately with a same solution volume of the 5′-adenylic acid, 5′-guanidylic acid, 5′-cytidylic acid, 5′-uridylic acid and cyclic adenosine triphosphate substrates (Sigma). A set of serial concentrations of substrates is 0.33, 0.338, 0.346, 0.354 and 0.362 mM. A volume of 2-µl cyclin-dependent kinase 1, protein kinase A and protein kinase C solutions is added. Enzymatic reactions are carried out under agitation at 20 °C. At an initial reaction interval of 0.5 h, an aliquot of 10-µl sample is siphoned off, diluted by saline (to 1:75) and put in a 1.5-ml diafiltration tube (Mw cut-off: 3.5 kDa). Dilution is treated by centrifugation. The reaction velocity is determined in terms of an inorganic phosphate group in the *Momordica charantia* protein phosphorylated counterparts, according to procedure for heat-digestion of counterparts, and characterization of the phosphate group by a phosphomolybdenum blue spectrophotometric method (*λ*=595 nm).

### 2.2 Dose-dependent susceptibility profiles and antifungal susceptibility testing

Antimycotics are analytical grade powders (Sigma-Aldrich, St Louis, MO). A concentrated stock solution is dissolved in dimethyl sulfoxide and diluted to the appropriate media for culture and test. A laboratory wild strain is *C. albicans* (Robin) Berkhout (ATCC^®^ 10231^™^). Sabouraud's dextrose agar (Oxoid) is the medium material. It is buffered to pH 7.0 prior to use. Acquisition of dose-dependent of susceptibilities of *C. albicans* is performed by gradually selecting to antimycotics. Antimycotics are loaded on the Sabouraud's dextrose agar, in the concentration ranges of 21.33–64.00 µg ml^−^^1^ (ketoconazole), 4.00–16.00 µg ml^−^^1^ (econazole), 3.77–11.30 µg ml^−1^ (miconazole), 21.33–64.00 µg ml^−^^1^ (5-flurocytosine), 1.33–4.00 µg ml^−^^1^ (nystatin), 2.67–8.00 µg ml^−1^ (amphotericin B) respectively. A set of culture media is divided into three parallels with antimycotics concentrations from the minimal to maximal extremes at the 1-fold interval. At first, fungal colony is grown on a commercial Sabouraud's dextrose agar plate for 48 h (at 35 °C). Fungal inoculum preparations are followed in procedure for the Clinical and Laboratory Standards Institute document M27-A2 [3], using a 0.5 McFarland latex standard (Izasa, SA) reference. It is adjusted with water to a typical turbidity range of 0.11–0.14 by means of the optical density determination at 530 nm. The fungal suspension is diluted by about 1:100 to get the enumeration of 1×10^3^ CFU ml^−1^ colony. Then a 100-μl aliquot of inoculum dilution is directed to per ml of the culture liquid with the minimal extreme concentration of antimycotics, and the fungus is cultured at 35 °C for 24 h. A volume of 100 µl fungal culture is treated by 100-fold dilution, followed by spreading the 100 µl-dilution on a Sabouraud's dextrose agar plate (with a same concentration antimycotics as that in the liquid medium) for 48-h culture. The plate count is enumerated and the color of fungal colony is recorded. After these steps, fungal colony is treated again by selection and screen procedure in another division for the antimycotics concentration at a high level, and the plate count and the colony color are recorded after culture. Three treatment repeats of fungus are achieved from the minimal to maximal concentrations of antimycotics.

Colony isolates in the final selecting repeat are used as fungal lineages for antifungal susceptibility testing. Broth microdilution susceptibility testing is performed according to the Clinical and Laboratory Standards Institute document M27-A2 microdilution procedure. Cell suspension is adjusted to match the turbidity of a 0.5 McFarland standard. It is then diluted in a testing medium and the dilution is added to each hole of the 96-well microtiter plates. A final inoculum concentration is 0.5×10^3^ to 2.5×10^3^ cells ml^−1^ (at an interval of 0.5×10^3^ cells ml^−1^). The concentrations of ketoconazole, econazole, miconazole, 5-flurocytosine, nystatin and amphotericin B are controlled to the 8-series levels from 0.083 to 10.624 µg ml^−^^1^ (at the 1-fold interval). Plates is incubated at 35 °C and read visually after 24 h. The minimal inhibitory concentration is defined as the lowest antimycotics amounts at which there is a significantly different response of fungal growth inhibition (at typical 5%). All experiments are done with triplicates and growth variations do not exceed 10%. A percentage of the susceptible dose-dependent *C. albicans* isolates are calculated at last.

### 2.3 Protein express determinations

Protein express is characterized with fungal collections in a large volume of cultures (200 ml Sabouraud's dextrose agar). Fungal collection is freeze-dried and a mass is obtained. Then the collection is dispersed and cell suspensions are ultrasonically disintegrated for 20 min (at 200-W power, using a work-to-break scale of 20 s). The disintegrated samples are centrifuged at 16,000 rpm for 30 min. Proteases in the supernatant are determined by a coomassie brilliant blue R-250 (CBB R-250) staining method (at a wavelength of 595 nm), in aid of the internal standard fitting of bovine serum albumin [2].

## References

[1] Qiao Y.B., Song L., Zhu C.C., Wang Q., Guo T.Y., Yan Y.H., Li Q.S. (2017). Development of a phosphorylated Momordica charantia protein system for inhibiting susceptible dose-dependent C. albicans to available antimycotics: an allosteric regulation of protein. Eur. J Pharm. Sci..

[2] Guo C.Z., Gao Y., Chang R.R., Yang L.R., Chu P.P., Hu X.M., Qiao Y.B., Li Q.S. (2015). Zirconium phosphatidylcholine-based nanocapsules as an in vivo degradable drug delivery system of MAP30, a momordica anti-HIV protein. Int. J. Pharm..

[3] H. John, D. Barbara, A. David, A. Beth, D. Steven, C. Vishnu, A. Mahmoud, E. Ana, C. Cynthia, O. Luis, A. Michael, J. Daniel, J. Thomas, In Clinical and Laboratory Standards Institute (CLSI), Reference Method for Broth Dilution Antifungal Susceptibility Testing of Yeasts, Approved Standard-Third edition, CLSI document M27-A3, (ISBN 1-56238-666-2), Clinical and Laboratory Standards Institute, 950 West Valley Road, Suite 2500, Wayne, Pennsylvania 19087, USA, 2008.

