# Supplementary material for: Dataset on preparation of the phosphorylated counterparts of a Momordica charantia protein for studying antifungal activities against susceptible dose-dependent C. albicans to antimycotics
Source: Data Brief. 2017 Sep 23;15:370–5. doi: 10.1016/j.dib.2017.09.041 (PMC5636016; doi:10.1016/j.dib.2017.09.041)
Supplement: Supplementary file 1 — Supplementary material [file mmc1.docx]

*Conflict of Interest Form*

The data in brief article “Dataset on preparation of the phosphorylated counterparts of *a Momordica charantia* protein for studying antifungal activities against susceptible dose-dependent *C. albicans* to antimycotics” by Yuanbiao Qiao, Li Song, Chenchen Zhu, Qian Wang, Tianyan Guo, Yanhua Yan, Qingshan Li (Manuscript Ref. No.: DIB-D-17-00768R2)

The data in this work is the Supplementary Material of the research article (Qiao et al., 2017 Eur J Pharm Sci. <https://doi.org/10.1016/j.ejps.2017.08.024>). Transparency data to this article can be found online at the site.

Besides, we reported no other conflict of interest form between authors.
